# Adherence and barriers to the vitamin D and calcium supplement recommendation at Danish nursing homes: a cross-sectional study

**DOI:** 10.1186/s12877-021-02719-4

**Published:** 2022-01-06

**Authors:** Charlotte Mortensen, Inge Tetens, Michael Kristensen, Pia Snitkjaer, Anne Marie Beck

**Affiliations:** 1 Department of Nursing and Nutrition, Faculty of Health, University College Copenhagen, Sigurdsgade 26, 2200 Copenhagen N, Denmark; 2 Department of Nutrition, Exercise and Sports, Faculty of Science, University of Copenhagen, Noerre Allé 51, 2200 Copenhagen N, Denmark; 3 Dietetic and Nutritional Research Unit, Herlev Gentofte Hospital, Borgmester Ib Juuls Vej 50, 2730 Herlev, Denmark

**Keywords:** Vitamin D, Calcium, Tablets, Nursing homes, Aged, Supplementation, Recommendation, Implementation, Adherence

## Abstract

**Background:**

Nursing home residents are in high risk of vitamin D deficiency, which negatively affects bone health. Vitamin D and calcium supplements haves shown to increase bone density and reduce fracture risk. Therefore, The Danish Health Authority recommends all nursing home residents a daily supplement of 20 μg vitamin D and 800–1000 mg calcium. However, adherence to the recommendation and knowledge of it is unknown. The aims of this study were to investigate adherence, knowledge, and potential barriers to this recommendation in Denmark.

**Methods:**

A cross-sectional electronic survey was conducted in May–June 2020 among 50 randomly selected nursing homes widely distributed in Denmark. Questions included degree of adherence to the recommendation at each nursing home as well as respondent’s knowledge and attitudes towards it, and experienced barriers in relation to adherence.

**Results:**

Respondents from 41 nursing homes answered the questionnaire, and these were mainly nurses (63%) or nursing home leaders (20%). Low adherence (≤ 40% of residents receiving both supplements) was reported at 35% of nursing homes, and only 8% of the nursing homes had a high adherence (> 80% of residents receiving both supplements). Most respondents (88%) had knowledge of the recommendation and 62% rated importance of increased implementation as high. Common explanations of low implementation were a lack of prescription by the general practitioner in the central electronic database (60%), resident-refusal to eat tablets (43%), chewing-swallowing difficulties (40%), and a high number of tablets given to the residents daily (34%).

**Conclusions:**

The recommendation of daily vitamin D and calcium supplements to Danish nursing home residents is poorly implemented even though knowledge of the recommendation is relatively high. Barriers relate to an ambiguity of responsibility between the general practitioners and the nursing home staff, as well as the high number of tablets to be consumed in total by the residents. These barriers must be targeted to improve adherence in this vulnerable group of institutionalized older adults.

## Background

Nursing home residents is a high-risk group when it comes to vitamin D deficiency [1–3] with prevalence rates depending on the chosen 25-hydroxyvitamin D cut-off to define deficiency [4]. The reasons include a poor appetite and the fact that few foods have a naturally high content of vitamin D, together with limited outdoor time and cutaneous synthesis of vitamin D [5]. Vitamin D deficiency in older adults negatively affects bone health [6] and physical performance [7], and leads to increased rate of falls [8]. One of the main functions of vitamin D in the body is to increase intestinal calcium absorption and thereby supporting bone density. A combination of vitamin D and calcium supplements to nursing home residents has shown to increase bone density and reduce fracture risk [6, 9]. Therefore, since 2010, the Danish Health Authority has recommended supplementation of 20 μg vitamin D and 800–1000 mg calcium daily to all 70+ years and all nursing home residents [10]. Likewise, vitamin D supplementation is recommended for older institutionalized adults in a variety of countries with the amount of vitamin D to administer varying worldwide [1, 11]. However, in spite of this, widespread vitamin D deficiency is seen among nursing home residents as well as poor implementation of the vitamin D recommendation through decades [1]. Until recently, little attention has been given to understand the underlying causes of this poor implementation or to find strategies to target it. However, a British study from 2020 showed that a limited use of vitamin D at nursing homes was explained by low awareness of the recommendation and different views on responsibility between professions. The latter related to the duality of the understanding of vitamin D as either medicine and the responsibility of the general practitioner (GP) or as dietary supplement and the responsibility of the care staff [12]. In Australia and Canada, understanding and targeting the barriers for implementing the vitamin D recommendation at nursing homes has been the subject of research, and mainly focusing on increasing knowledge among the care staff [13, 14]. When it comes to research among older adults in Denmark, studies are mainly conducted among community dwelling [15]. However, we still lack studies among institutionalized older adults and the degree to which the recommendation is implemented at Danish nursing homes. Moreover, it is unknown what knowledge the care staff have of the recommendation, as well as which potential barriers for improved implementation may exist.

The aims of this study were to evaluate the current implementation, practice of documentation, knowledge, attitudes, experienced barriers, and suggested solutions in relation to the vitamin D and calcium supplement recommendation at Danish nursing homes.

## Methods

### Development and distribution of questionnaire

This cross-sectional study was carried out using an electronic 20-items questionnaire (Enalyzer software) developed for the study. The questionnaire was qualified by e-mail correspondences with the Danish Patient Safety Authority, who carries out supervision in health and older adults, and the General Practitioner’s Organization (PLO) in the Capital Region. Subsequently we pilot tested it at a Danish nursing home according to its comprehensibility and content validity and adjusted before distribution. Nursing homes were identified at a website containing an overview of all nursing homes (www.plejehjemsoversigten.dk). From each of the five geographical regions in Denmark, we randomly selected 10 nursing homes of variable size to ensure diversity in both geographical location and number of residents. Nursing home leaders were contacted by telephone, and recruitment finished when 50 nursing homes had agreed to participate. There were no exclusion criteria. During the telephone conversation the nursing home leader was asked to identify an employee to answer the questionnaire, and it was specified by the investigator, that the preferable respondent was one engaged in medication. Subsequently, a link to the questionnaire was send by e-mail to the respondent selected by the nursing home leader. A reminder was sent after two-three weeks if the questionnaire was not answered.

### Background data

Background data included geographical region of the nursing home, size based on number of residents, job title of respondent, and whether the nursing home had an affiliated GP.

### Topics

The questionnaire consisted of both open-labelled questions and questions with predefined categories and topics fell into five categories. *I) Degree of implementation:* Respondents categorized the percentage of residents receiving vitamin D and/or calcium supplements as well as multivitamins (the latter recommended to small-eating older adults [16]) in any dose ≥ five days/week according to the categories 0%, 1–20%, 21–40%, 41–60%, 61–80%, 81–99, 100% and ‘unknown’. *II) Practices of documentation:* Initial desk research revealed that nursing homes have varying documentation practices before providing the supplements, which relates to different views on responsibility for dietary supplements. In Denmark, a central electronic database; the Common Medicine Card (CMC) exists for the GP’s to prescribe medicine [17]. However, since vitamin D and calcium are dietary supplements and not medicine, it is not recommended to use the CMC. Instead the Danish Patient Safety Authority recommends that the care staff document supplementation in their own electronic care record system at each nursing home [18, 19]. Despite this, some Danish municipalities prohibits administration of supplements not in CMC ([20]; personal communication chair of PLO in the Capital Region). Since an understanding of documentation practices are vital when investigating barriers, we asked to the practice used at each nursing home. Moreover, we asked respondents if they were prohibited to administer supplements not prescribed in CMC by their municipality. *III) Knowledge and attitude:* Respondents were asked if they had knowledge of the recommendation and if yes whether they knew the recommended doses. Moreover, they rated own perceived knowledge on health effects of supplementation and prioritization of improved implementation, on a scale from 1 to 5 with 1 rating minimal and 5 rating maximal knowledge/prioritization. In addition, we asked respondents to state if and which specific vitamin D health effects they knew as free-text statements. *IV) Experienced barriers:* Based on initial research we listed potential barriers for not providing the residents with the supplements, such as chewing-swallowing difficulties among the residents or lack of knowledge of the recommendation among the staff. Respondents were asked to choose which barriers they viewed as most significant. *V) Suggestions for improved implementation:* Based on initial research, potential strategies to improve implementation were listed, such as deprescription of unnecessary medication or improved knowledge among the staff. Respondents were asked to prioritize them on a 1–5 scale with 1 being least and 5 most important.

### Statistical analyses

Data in the online questionnaire were recoded in Microsoft Excel by the investigator and were analyzed using the statistical software IBM SPSS Statistics 25. Descriptive statistics are presented as numbers and frequencies. During data analyses, the outer categories of percentage of residents given supplements were merged due to few answers in the categories 0 and 100%. Degree of adherence was defined as either *low*; ≤ 40%, *medium*; 41–80% and *high*; > 80% receiving supplements, the latter cut-off also suggested by others [21]. The 1–5 scale categories used to assess knowledge and prioritization were merged leaving three categories: 1–2, 3 and 4–5.

## Results

### Background data

A total of 55 nursing homes were contacted by telephone until 50 nursing homes agreed to participate (participation rate 91%). Among the 50 nursing homes receiving the questionnaire, a total of 41 nursing homes answered the questionnaire (one per nursing home, response rate 82%)) through the link in May-June 2020. The nursing homes were evenly distributed across the country (seven to 10 from each of the five regions) and with a mean of 51 residents (min 22, max 96). Of these 41 responders 34 completed the entire questionnaire (resulting in *n* from 34 to 41 depending on question). Most respondents were nurses (63%), followed by nursing home leaders (20%), social-and-health-care assistants (8%), clinical dieticians (5%), matrons (2%) and meal coordinators (2%). A GP was affiliated at 66% of the nursing homes.

### Degree of implementation

Questions concerning implementation of the Danish Health Authority recommendation were answered by 37 nursing homes. Our data showed that 35% of the nursing homes had a low adherence, i.e. providing ≤40% of their residents with *both* vitamin D and calcium ≥ five days/ week, whereas 8% had a high degree of adherence of > 80% (Table 1). Nearly one fifth of the respondents stated that they did not know the degree of adherence at their nursing home. When it comes to giving their residents *either* vitamin D *or* calcium this was even less frequently practiced than both supplements together. Concerning the recommendation of giving multivitamins to residents, 29% of the nursing homes had low adherence, and 8% had high adherence, whereas 14% did not know degree of adherence (Table 1).

**Table 1:** Adherence shown as number of nursing homes providing supplements to a given percentage of residents

			Vit. D + calcium	Vit. D alone	Calcium alone	Multi-vitamins
Percentage of residents	Adherence^a^		*n* (%)	*n* (%)	*n* (%)	*n* (%)
	*Low*	0–20%	10 (27)	17 (46)	20 (54)	4 (11)
		21–40%	3 (8)	5 (13)	3 (8)	7 (18)
	*Medium*	41–60%	9 (24)	4 (11)	4 (11)	13 (35)
		61–80%	5 (14)	1 (3)	2 (5)	5 (14)
	*High*	81–100%	3 (8)	1 (3)	0 (0)	3 (8)
		Unknown	7 (19)	9 (24)	8 (22)	5 (14)
		Total	37 (100)	37 (100)	37 (100)	37 (100)

Values are presented as n (%), *n* = 37

^a^ Degree of adherence is defined as *low*; ≤ 40%, *medium*; 41–80%, and *high*; > 80% of residents given supplements

### Documentation practices

The practices of documentation at the nursing homes before vitamin D and calcium were given to the residents are shown in Table 2. Multiple choices were allowed. It was most common that the supplements were prescribed by the GP in the Common Medicine Card, CMC – either by *GP’s own initiative*, or *after request* from the nursing home staff. At only one fifth of the nursing homes the staff made a request to the GP to give the supplements and subsequently used own internal journaling system to document supplementation (Table 2). In addition, according to half of the respondents, their municipality prohibited administration of dietary supplements unless the GP prescribed it in CMC.

**Table 2:** Documentation practices for vitamin D and calcium supplementation at nursing homes

	***n*** (%)
GP took initiative for supplementation and prescribed in CMC	29 (83)
Nursing home took initiative for supplementation and GP prescribed in CMC	22 (63)
Nursing home took initiative for supplementation and documented in internal system after accept from GP	7 (20)
Dietary supplements neither documented in CMC nor internal care record system	2 (6)

*n = 35.* Multiple choices possible, *abbreviations: GP* General Practitioner, *CMC* Common Medicine Card (The central electronic database for prescription of medicine)

### Knowledge of and attitudes to the recommendation

Regarding knowledge of the Danish Health Authority recommendation, 12% did not know the existence of a recommendation of vitamin D and calcium supplementation for nursing home residents, whereas 37% knew of the recommendation but not the exact doses. The remaining 51% were aware of both recommendation and doses. These results were confirmed when asked about which doses and names of supplements were given, as almost half of the respondents (47%) were not able to recall any specific doses or names of supplements.

**Fig. 1:**
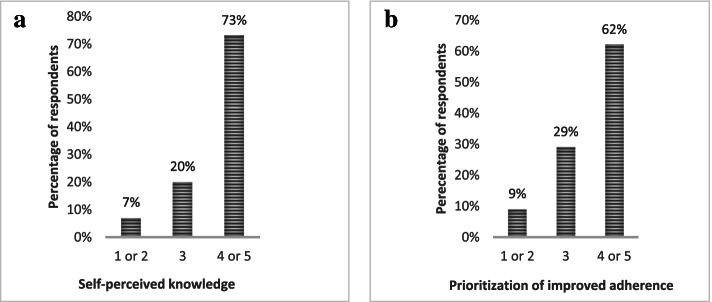
**a** Percentage of respondents (*n* = 41) rating their self-perceived knowledge of health effects of vitamin D on a scale, where 1 corresponds to *lack of knowledge* and 5 corresponds to *substantial knowledge*. **b** Percentage of respondents (*n* = 35) rating their prioritization of improving adherence to the recommendation on a scale where 1 corresponds to *not important at all* and 5 to *extremely important*

Respondents generally viewed their own knowledge of potential health effects of the supplementation as high, defined as level 4 or 5 on the 1–5 scale (Fig. 1a). When asked to give specific examples of vitamin D health effects, the mentioned health effects could be categorized as follows: I) prevention of osteoporosis / falling (*n* = 27); II) better muscle function (*n* = 10); III) improved immune system (*n* = 9); IV) lower risk of depression (*n* = 5); and V) more energy (*n* = 2). In addition, respondents generally viewed it as important to work with an improved implementation of the recommendation (Fig. 1b).

### Barriers and solutions

**Fig. 2:**
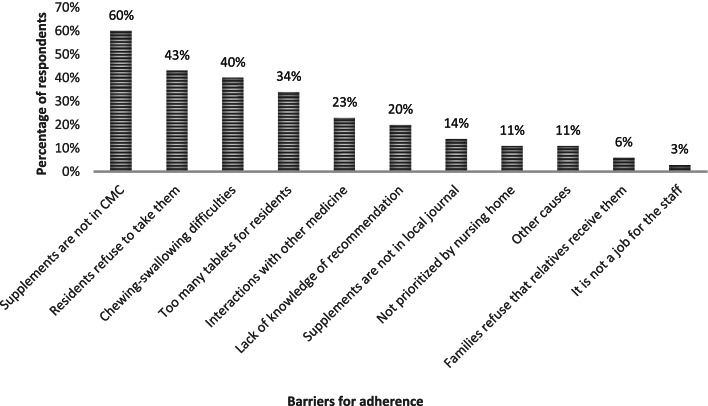
Percentage of respondents choosing a specific cause for not giving the supplements to all residents, multiple choices possible, *n* = 35 (predefined categories). *Abbreviations*: CMC; Common Medicine Card (The central electronic database for prescription of medicine)

The most frequent cause of not providing all residents with the supplements was a lack of a prescription by the GP in CMC (Fig. 2). Other important causes related more directly to the residents as refusal to eat the tablets, chewing-swallowing difficulties, and omission of the tablets due to a high number of tablets already. In addition, more than 20% of respondents stated interactions between the supplements and medicine as a reason for non-adherence (Fig. 2).

When asked to rate six suggested strategies for improved adherence using a 1–5 scale of importance, four of the solutions were prioritized as 4–5 among most respondents (Table 3). The two highest-rated solutions were that the GP should prescribe the supplements in CMC and that the medication lists should be reviewed to de-prescribe unnecessary medication. The third-most preferred solution was more knowledge among the staff, followed by the increased use of chewing tablets, droplets, or sprays (Table 3).

**Table 3:** Preferred solutions for improved adherence to the recommendation

	***n*** (%)
GP should prescribe the supplements in CMC	*28* (82)
The medication lists should be reviewed to de-prescribe unnecessary medication	*28* (82)
Nursing home staff needs more knowledge about the recommendation	*26* (76)
The supplements should be given as chewing-tablets, droplets, or sprays	*22* (65)
Nursing home should take initiative and get accept from GP to give and document supplementation in internal care record system	*11* (32)
The families of the residents should be included	*10* (29)

Values are presented as *n* (%). *n* = 34. Data show number of respondents rating each of six given solutions 4 or 5 on a 1–5 scale where 1 corresponds to not important at all and 5 to extremely important. *Abbreviations*: CMC; Common Medicine Card (The central electronic database for prescription of medicine), GP; General practitioner

## Discussion

In this cross-sectional study, respondents from 41 Danish nursing homes answered a questionnaire on adherence to the vitamin D and calcium recommendation. We found that only 8% of the participating nursing homes stated a high degree of adherence. Likewise, adherence to the recommendation of giving multivitamins daily, which may contain as little as 5 μg vitamin D per tablet, was poorly implemented. A substantial barrier for following the vitamin D and calcium supplement recommendation was lack of prescription by the GP in the electronic database, CMC, even though 66% of nursing homes had own GP affiliated. This is interesting since it is advised that the GP’s do not use the CMC for prescribing dietary supplements in Denmark [18]. Instead the care staff should document supplementation in the internal electronic care record system [18, 19]. Even though the care staff are formally allowed to take responsibility of dietary supplementation as per national guidelines, our data show that they prefer the responsibility to lie with the GP’s since the preferred strategy for improved implementation was having the GP’s to prescribe the dietary supplements in CMC. Moreover, a strategy with low priority was leaving it to the care staff to contact the GP. These preferences can also partly be explained by the fact that half of the nursing homes in this study stated that their municipality did not allow them to administer supplements not prescribed in CMC.

To our knowledge, this study is the first to evaluate the degree of adherence to the vitamin D and calcium recommendation at Danish nursing homes. A previous study focused on community-dwelling Danish + 70-year-olds, among which 32% followed the recommendation [15]. Our data suggest that adherence is also poor among Danish nursing home residents. This is supported by unpublished data from a study in which 57% of residents at 27 nursing homes in the region of Southern Denmark had both vitamin D and calcium supplements (correspondence with first author [22]). Another Danish study has shown that among hip fracture patients (+ 65 year-olds) living in nursing homes or sheltered housings, 59% did not receive vitamin D supplementation upon admission [23]. Internationally, studies find variable but generally poor degree of adherence. Among Australian age care residents, adequate vitamin D (≥ 20 μg/day) and calcium (≥ 500 mg/day) supplement use were seen in 56 and 18% of residents, respectively [13]. Among American nursing homes residents, 7% received 20 μg vitamin D and 40% received 1000 mg calcium [24], whereas another study showed prescription of vitamin D among 35% of nursing home residents [21]. Among Canadian nursing homes residents, 30–40% had adequate vitamin D and calcium supplement intake [14]. Our data show that this failure to adhere to the evidence-based recommendation is also seen in Denmark. Of course, it should also be noted, that not all residents should be offered vitamin D and calcium supplementation such as individuals with elevated blood levels of calcium or phosphate [25]. But even with this in mind, adherence to the recommendation is generally low.

In line with our findings, a British study by Fallon et al.*,* 2020, found a generally positive attitude towards giving vitamin D supplements among health care professionals [26]. In addition, Williams & Williams, 2020, reported that it is a very common view among British public health professionals that vitamin D is the responsibility of the GP’s [12]. According to their interviews the supplement was only given on individual basis when prescribed by a GP in response to fall, fractures, vitamin D deficiency, or osteoporosis but never used routinely [12]. In line with this, a Belgian survey showed that only 55% of GP’s with patients in nursing homes systematically prescribed vitamin D [27]. Our data indicate that similar views, practices, and barriers exist in Denmark. Thus, as also concluded by Williams & Williams, it seems that a paradigm shift is needed, so vitamin D is viewed both as a protective nutrient and as medicine to improve implementation.

Our data also showed, that when nursing homes do not adhere to the recommendation, it is often explained by a considerable number of tablets daily and difficulties with chewing and swallowing them. A Danish study reported prescription of a median of eight drugs among more than 5000 nursing home residents [28]. In line with this, the second-most preferred strategy to improve implementation in our study was reviewing medication lists to avoid unnecessary tablets. Another preferred potential solution among our respondents was use of chewing tablets, droplets, or sprays, the latter two only for vitamin D. Of these, only chewing tablets were already used at some of the nursing homes. Even though the bioavailability of vitamin D with an oil-soluble vehicle has previously shown to exceed that of powder-based vehicles [29], vitamin D droplets in Denmark are marketed infants who are recommended daily vitamin D droplets, which may explain that these products are not widespread at nursing homes. Vitamin D sprays are also sold at the pharmacies and previously it has been shown that an oral spray was equally effective to capsules in increasing vitamin D status in healthy adults [30]. Thus, use of droplets or spray for institutionalized adults could be a promising strategy to overcome chewing and swallowing difficulties. In continuation of identifying reasons for low adherence, critical elements for a successful implementation are strategies which may be conceived as acceptable, feasible and appropriate for the nursing homes. Thus, these elements from implementation research focusing on bridging the gap between evidence-based practices and its uptake [31] should be considered in future phases of the study.

The failure to implement the evidence-based recommendation at nursing homes can have considerable health consequences since vitamin D deficiency among institutionalized older adults is associated with increased risk of dementia [32], frailty [33], falls [34], and mortality [35, 36]. Vitamin D supplementation has shown to reduce the rate of falls [8], be beneficial on immune function [37] and prevent acute respiratory infections [38], and a combination of calcium and vitamin D to institutionalized older adults to increase muscle strength [39] and reduce the risk of fractures [9, 34]. Thus, with the above evidence in mind, we hypothesize that targeting the barriers for the vitamin D and calcium supplement recommendation could improve physical functioning and life quality for Danish nursing home residents. However, in this regard, it is of course also relevant to consider the overall nutritional status of the residents and its potential impact on physical functioning. Previous Danish research has shown that nursing home meals seldom or never met nutritional recommendations when it came to energy and macro nutrient content [40] and it may be vital also to correct for instance sub optimal energy intake in order to see an effect of micronutrient supplementation. In addition, another micronutrient, magnesium, may be important to consider due to its role for normal vitamin D physiology [41].

This cross-sectional study has some limitations. We emphasized that the preferred respondent should be one engaged in medication. However, as 14–24% of respondents answered “unknown” to degree of adherence it can be questioned if the most relevant respondents answered, and moreover, if more valid data could be obtained by asking respondents to look in the journal of each resident. As data collection took place during the coronavirus pandemic, nursing homes were struggling with changing guidelines, and this may have contributed to the relatively high number of “unknown” answers. However, as other parts of the questionnaire show a certain knowledge of potential health effects of vitamin D, we assume that relevant respondents were generally chosen. Moreover, we are aware of the possibility of social desirability bias which could lead to overestimation of degree of adherence, priority of improved adherence and self-perceived knowledge. It should also be noted that most questions had predefined answers, and we cannot rule out if more open answers would affect the results. For instance, adherence was reported according to predefined intervals as we did not expect the staff to be able to report an exact frequency of adherence. In addition, we simplified questions on adherence by asking to “any dose” and not specifically the recommended doses, and we cannot know if nursing homes provided the recommended doses. Strengths of the present survey include that questions and categories of answers were developed after contact with stakeholders and pilot tested at a nursing home. This increases the chance that the questionnaire addressed the most relevant issues related to the topic. In addition, we expect only a limited bias of volunteering since the response rate was quite high. Moreover, even though our relatively small sample size constitutes 5% of Danish nursing homes, we ensured a geographical diversity as well as nursing homes of variable size. Based on this, we find that results can be transferred to Danish nursing homes in general.

## Conclusions

In this cross-sectional study, we found that only 8% of Danish nursing homes have a high degree of adherence to the recommendation of providing residents with vitamin D and calcium supplements. Our data suggest two main barriers: One relates to the ambiguity of responsibility between the GP’s and the care staff at the nursing homes, and another to the relatively high number of tablets consumed daily by the residents. Studies targeting these barriers are needed to improve adherence for this vulnerable group of institutionalized older adults.

## Data Availability

The datasets used and/or analysed during the current study are available from the corresponding author on reasonable request.

## Abbreviations

CMC: Common Medicine Card
GP: General practitioner
PLO: General Practitioner’s Organization
